# Editorial: Impact of coronavirus disease 2019 (COVID-19) pandemic on nosocomial infection

**DOI:** 10.3389/fmed.2023.1298645

**Published:** 2023-10-13

**Authors:** Mingke Wang, Mahlagha Dehghan, Chunhui Li, Amedeo Amedei, Alfonso J. Rodriguez-Morales

**Affiliations:** ^1^Naval Medical Center of PLA, Naval Medical University, Shanghai, China; ^2^Nursing Research Center, Kerman University of Medical Sciences, Kerman, Iran; ^3^Department of Infection Control Center, Xiangya Hospital, Central South University, Changsha, China; ^4^Department of Experimental and Clinical Medicine, University of Florence, Florence, Italy; ^5^Faculty of Health Sciences, Universidad Científica del Sur, Lima, Peru; ^6^Gilbert and Rose-Marie Chagoury School of Medicine, Lebanese American University, Beirut, Lebanon

**Keywords:** COVID-19, SARS-CoV-2, nosocomial infection, clinical characteristics, outcome, pathophysiologic mechanisms, prevention, treatment

Globally, up to September 1, 2023, there have been 770,085,713 confirmed cases of coronavirus disease 2019 (COVID-19) caused by severe acute respiratory syndrome coronavirus 2 (SARS-CoV-2), including 6,956,173 deaths, as reported to World Health Organization (1). Many studies have arisen in response to the COVID-19 pandemic. However, clinical characteristics, outcomes, the underlying pathophysiologic mechanisms, and effective prevention and treatment measures of nosocomial infections (NIs; also called healthcare-associated infections) associated with COVID-19 are still unclear in many ways. Meanwhile, the NIs control system should continuously improve even in the post-COVID-19 era. Therefore, this Research Topic was proposed, and it aimed to collect manuscripts on recent progress and future perspectives on COVID-19 that help promote the development of effective prevention and treatment measures of COVID-19-associated NIs in different departments of the hospital, explore the changes in NIs pre- and post-COVID-19, and improve the quality of NIs control. Six papers have been accepted in this Research Topic, including one brief research report and five original research articles.

In the study reported by Huang et al., the authors compared the observation indicators of NIs and their changing trends at a large tertiary maternity hospital before and during the COVID-19 pandemic, and found that the prevention and control measures for the COVID-19 pandemic have reduced the NIs, mainly respiratory, gastrointestinal, and catheter-related infections. Better management of antibiotic use, better training for physicians, better environmental hygiene, hospital-specific strategies to guide clinical departments in their COVID-19 prevention efforts, widely used masks coupled with adequate social distancing, and diligent hand hygiene may have reduced the spread of hospital pathogens. However, Su et al. found that the prevalence of NIs decreased in most departments after the COVID-19 pandemic, except for the ICU, mainly regarding respiratory, gastrointestinal, and oral infections. In contrast, bloodstream and catheter-related infections did not differ (2). These results may be due to different nosocomial pathogens and prevention and control measures for the COVID-19 pandemic in different hospitals.

Many studies have demonstrated that improving hand hygiene compliance (HHP) significantly reduces NIs (3–5). However, Makhni et al. (6) suggested that high HHP was possible yet difficult to sustain. In addition, Moore et al. (7) found that HHP first increased as the pandemic began and then decreased as it progressed. In this Research Topic, Zhang et al. developed a relatively perfect hand hygiene monitoring system. They evaluated the HHP rate in a tertiary hospital in China before and after the COVID-19 outbreak. They documented that reward and punishment mechanisms, increasing hand hygiene facilities, diversified hand hygiene publicity and training and two levels of supervision and assessment could improve the HHP rate in hospitals. More hand hygiene improvement strategies are needed, and implementing these strategies to build a robust system is also of utmost importance (8).

In another study, Luo et al. designed a self-designed questionnaire used to estimate basic information, work experience, and status of infection prevention and control (SIPC) of 269 emergency support frontline healthcare workers (ESFHCWs). They evaluated the factors influencing the SIPC of these ESFHCWs under closed-loop management in a provincial-level tertiary hospital treating COVID-19 patients in the Xinjiang Uygur Autonomous Region. The authors found that the closed-loop management strategy may effectively reduce the infection rate of hospital-acquired COVID-19 among ESFHCWs, and work seniority, anxiety disorder, and consumption of other medications were the independent risk factors influencing COVID-19 prevention and control status. These results provided some selection criteria for ESFHCWs in COVID-19 medical assistance.

The SARS-CoV-2 test strategy can support analyzing the ongoing spread of communicable diseases and could be a valuable tool for infection prevention control in hospitals (9). Evans et al. reported an individual-based model parameterised using multiple datasets, simulated the transmission of SARS-CoV-2 to patients and HCWs between March and August 2020 and evaluated the impact of different testing strategies for the detection of nosocomial COVID-19 in English hospitals. Although testing all patients on admission and retesting after 3 or 5 days increased the proportion of nosocomial cases detected by 9.2%, and discharge testing increased detection by a further 1.5%, no significant difference in the rates of nosocomial transmission between testing strategies was observed even the turnaround time of the test increased. This study provided insight into the efficacy of testing strategies through data-driven modeling, and more effective data-driven test strategies should be recommended for better response during the pandemic (10).

The knowledge, attitudes, and practices (KAP) of HCWs toward COVID-19 could affect their compliance with prevention and control activities during the pandemic (11). Inadequate knowledge and incorrect attitudes among HCWs can influence their practices and lead to delayed diagnosis, poor infection control practices, and the spread of COVID-19 (12). Sun et al. explored the KAP of HCWs in the radiology department toward the prevention and diagnosis of COVID-19 in a multicenter cross-sectional study and documented that radiology medical workers showed moderate knowledge but good attitudes and practices of COVID-19 prevention and diagnosis. Additionally, knowledge was positively correlated with attitude that was positively correlated with practice, and attitudes significantly mediated the association between knowledge and practice.

Finally, Nasoufidou et al. investigated the potential correlation between length of hospitalization and COVID-19 patients' clinical laboratory data of admission, including the total severity score (TSS) from chest computed tomography based on a retrospective study conducted in Greece. By multivariate analysis, they found that age and TSS were significantly and positively correlated with hospitalization time. Thus, based on the age and TSS of the patients at admission, healthcare providers can initially judge the severity of COVID-19 patients, identifying those with a potentially high risk of prolonged hospitalization. That may be useful for healthcare service resource allocation and decision-making processes in further patient management.

Overall, several studies covered in this Research Topic have shown that the impact of COVID-19 on NIs is extensive, including short-term and long-term effects, as well as an impressive impact on HCWs and patients. For COVID-19 management, proactive prevention strategies implemented in the hospital (13), global and regional evidence-based guidelines are needed (14) and new therapeutic approaches (15, 16). Indeed, more studies are still required to understand the relationship between the COVID-19 pandemic and NIs and ultimately decrease the occurrence of NIs effectively.

## Author contributions

MW: Conceptualization, Writing—original draft, Writing—review and editing. MD: Writing—review and editing. CL: Writing—review and editing. AA: Writing—review and editing. AR-M: Conceptualization, Writing—review and editing.
